# Person-centered abortion care in public health facilities across four regions of Ethiopia: a cross-sectional quantitative study of client experiences

**DOI:** 10.3389/frph.2024.1331682

**Published:** 2024-09-04

**Authors:** Bekalu Mossie Chekol, Sarah McCaffrey, Sally Dijkerman, Valerie Acre, Demeke Desta Biru, Abiyot Belai Mehary, Samuel Muluye

**Affiliations:** ^1^ Ipas Ethiopia, Addis Ababa, Ethiopia; ^2^ Mailman School of Public Health, Columbia University, New York, NY, United States; ^3^ Ipas United States, Durham, NC, United States

**Keywords:** abortion, comprehensive abortion care, Ethiopia, public health facilities, service quality, quality of care, client perspective, person-centered care

## Abstract

**Introduction:**

Ethiopia has made remarkable progress in expanding access to and provision of comprehensive abortion care. However, complications due to unsafe abortion persist. As efforts to increase quality of comprehensive abortion care continue, evaluating service quality is critical. Although “women-centered” abortion care is a central component of Ethiopia's technical guidelines for safe abortion, research has mostly focused on access to care, availability of services, and meeting clinical criteria, rather than examining service quality from abortion clients’ perspectives. This study assesses the quality of comprehensive abortion care (CAC) in public health facilities, from clients’ perspectives, in four regions of Ethiopia to examine how person-centered care differs based on facility and service characteristics.

**Methods:**

We conducted 1,870 client exit surveys in 2018 using structured questionnaires with women who received induced abortion or postabortion care services from 76 public health facilities across four regions: Tigray, Amhara, Oromia, and Southern Nations, Nationalities, and People's. We operationalized person-centered care by mapping 30 indicators of quality to five of the six domains in the Person-Centered Care Framework for Reproductive Health Equity developed by Sudhinaraset and colleagues (2017): dignity & respect; autonomy; communication & supportive care; trust, privacy, and confidentiality; and health facility environment. We calculated descriptive, bivariate, and multivariable statistics to examine associations between service characteristics and person-centered care.

**Results:**

CAC clients reported high levels of person-centered care, with exceptionally positive experiences for outcomes in the dignity and respect and trust, privacy, and confidentiality domains. However, there was notable room for improving client experiences across three domains: autonomy, communication and supportive care, and health facility environment. Client-reported quality outcomes differed significantly by diagnosis (induced or postabortion care), region, health facility type, and procedure type. Clients in Amhara, clients at tertiary and primary hospitals, and clients who received postabortion care reported lower levels of person-centered care.

**Discussion:**

The positive experiences reported by comprehensive abortion care clients bolster evidence of the impact of the Ethiopian government's strategy to increase abortion access in the public health sector. However, notable disparities exist for key subgroups, particularly those seeking postabortion care and people visiting tertiary and primary hospitals. Quality improvement efforts should concentrate on improving abortion clients’ autonomy, communication and supportive care, and the health facility environment. The Ethiopian Ministry of Health and its partners must dedicate resources to improve postabortion care quality, integration of reproductive health services within CAC, and pain management for MA clients as vital interventions.

## 1 Introduction

Ethiopia is considered to have a “semi-liberal” abortion law (1). The current law allows for abortion in cases of rape, incest, incurable fetal deformity, if continuation of the pregnancy or birth of the child endangers the life of the pregnant person or the child, if they are mentally or physically disabled, or if they are a minor who is physically or mentally unprepared for childbirth, and in case of grave and imminent danger which can be averted by an immediate intervention (2). Since the liberalization of the abortion law in 2005, Ethiopia has achieved remarkable progress in improving access to and provision of safe abortion services. The Ethiopian Ministry of Health issued the “Technical and Procedural Guidelines for Safe Abortion Services in Ethiopia” in 2014 (3) which detailed the ability of public health facilities to provide safe induced abortion and postabortion care (PAC)^1^ services that center choice, accessibility, and quality. As a result, the public sector has become increasingly important in the provision of comprehensive abortion care (CAC)^2^, with national studies documenting an increase in public sector-provided abortion care from 36% in 2008 to 56% in 2014 (4). Concurrently, investments in training mid-level providers and the expansion of medical abortion (MA) have contributed to significant improvements in maternal health outcomes (4–7). The maternal mortality ratio for Ethiopia has declined from 865 per 100,000 live births in 2005 to 401 per 100,000 live births in 2017 (8), and unsafe abortion is no longer considered to be a leading cause of maternal deaths (9).

The importance of high-quality health care services, both as a mechanism to encourage care-seeking and improve human rights, is well-established (10, 11). The 2022 World Health Organization (WHO) abortion care guidelines defined the six components of quality as follows: effective, efficient, accessible, acceptable/person-centered, equitable, and safe (12). Prior studies have elucidated the relationship between low quality of care with high levels of abortion stigma and increased abortion-related morbidity and mortality, indicating that quality improvement interventions are important for reducing community stigma and improving health outcomes (13–15). Additional benefits include increased knowledge and uptake of contraceptive methods (16, 17).

The Person-Centered Care Framework for Reproductive Health Equity developed by Sudhinaraset et al. (18) has been adapted for CAC and provides a roadmap for effectively evaluating client experiences. The framework lays out six domains: dignity & respect; autonomy; communication & supportive care; trust, privacy, and confidentiality; social support; and health facility environment (19). Altshuler and Whaley (20) used this framework for a comprehensive review of person-centered abortion care from diverse country settings and unfortunately found that health facilities and providers often fail in providing adequate person-centered care to CAC clients (20). This results in devastating impacts for those seeking induced abortion or PAC, including negative mental health and psycho-social outcomes, delayed care-seeking, and using unsafe methods to avoid going to health facilities (14, 15, 20). These consequences emphasize the importance of dedicating resources to evaluate and improve person-centered abortion care.

Acceptable, person-centered care is often overlooked by evaluators (10, 21). A systematic review of abortion service quality indicators found that while advances have been made, most indicators still focused on infrastructure and technical competence of providers, with far fewer examining the experience of clients related to provider-client interaction, decision-making, or provision of information (22). This holds true in Ethiopia, despite “women-centered abortion care”^3^ being a central component of the country's technical guidelines (3). Research has mostly focused on access to care, availability of services, and meeting clinical criteria, rather than examining quality of CAC services from abortion clients’ perspectives (7, 17–19). For example, McMahon and colleagues evaluated national availability of PAC services in 2020 using the presence of essential resources and supplies to perform PAC signal functions (e.g., uterine evacuation) at health facilities. Despite progress toward universal PAC availability at health facilities (65%–70% of facilities providing PAC), 1 in 10 hospitals and 1 in 4 health centers that reported providing PAC lacked the signal functions required to meet minimum clinical standards (18). This finding suggests serious potential ramifications for people seeking abortion care at these facilities in terms of both clinical and person-centered quality.

Recent research in Ethiopia has largely left integral aspects of CAC quality—notably, person-centered care quality—to be insufficiently examined (23, 24). It is widely understood that access to health services does not necessarily mean that services are of high-quality (25, 26), and this remains true for CAC services (10). When person-centered care is evaluated, it is often accomplished using questions that employ broad statements about client satisfaction. Yet, due to stigma, lack of confidentiality, or gratefulness for being provided the abortion procedure, findings of satisfaction are often universally high and do not tend to differ based on demographic or service characteristics (10). For example, a study from Ethiopia in 2005 evaluating quality of PAC in government hospitals in Addis Abba found that 92.3% of patients reported satisfaction with services (27). However, in-depth studies analyzing CAC from the client perspective have demonstrated that when induced abortion and PAC clients are asked about specific aspects of care, there is greater variability in responses (19, 20, 28, 29). Similarly, Mossie Chekol et al. (28) found differences in patient satisfaction among CAC clients in Addis Ababa with regards to the abortion procedure type and facility type, with higher satisfaction found for manual vacuum aspiration (MVA) and public health facilities compared to MA services and private facilities, respectively. This study has been instrumental in painting a clearer picture of abortion clients’ experiences, but more research is necessary to provide a comprehensive depiction of person-centered CAC in rural areas and to understand differences by public health facility level, diagnosis, and region.

As investments in the public sector to increase CAC access have expanded in Ethiopia, analyzing quality of CAC services and centering patient experiences provides a key opportunity to further improve the health outcomes of women and girls (15, 25). Building upon person-centered abortion care frameworks utilized in Kenya and prior research within Ethiopia (19, 28, 30), this study utilizes client perspectives to examine the quality of induced abortion and PAC services—specifically, person-centered care, in public health facilities across four regions of Ethiopia [Tigray, Amhara, Oromia, Southern Nations, Nationalities, and People's (SNNP)]. Through assessing the differences in person-centered care based on facility and service characteristics, this research aims to inform health system interventions with the goal of improving the quality of CAC across Ethiopia.

## 2 Methods

### 2.1 Study design, setting, and population

Our objective was to examine the quality and variability of person-centered care for people seeking CAC services across facility and service characteristics in Ethiopian public health facilities. We employed a cross-sectional multi-stage stratified sample survey design using structured client exit interview (CEI) questionnaires. We conducted this research between November 2018 and March 2019. The research protocol and data collection instruments were reviewed and approved for adherence to ethical standards by the Ethiopian Public Health Institute (EPHI) Scientific and Ethical Review committee.

The research setting included thirty-two zones located within four regions of Ethiopia: Tigray, Amhara, Oromia, and SNNP. These four regions were selected to be included in the study because of their mixture of urban and rural areas and socio-demographic diversity. Inclusion of these four large regions allows for increased generalizability because together they comprise the majority, over 80%, of the Ethiopian population (31) and, similarly, an estimated 79% of all abortions from the latest regional estimates (6).

The study population included people who met the following eligibility criteria: received an induced abortion or PAC service, in stable health condition, above the age of 13, and consented to participate in the research study. For minors under the age of 18 parental or guardians consent was obtained for their participation in this study, though they are legally permitted to seek sexual and reproductive health (SRH) services without the consent of parents or guardians.

### 2.2 Sampling procedure

We used a stratified sampling approach to select health facilities, each facility serving as one cluster. A list of all public health facilities offering PAC and/or induced abortion services in the 32 zones within the four regions served as the sampling frame, which we then partitioned into strata using three levels of stratification: region, zone, and facility type (hospital/health center). Overall, the stratification generated 128 strata. From each stratum, a health facility was selected randomly. The number of clients recruited from each sampled health facility, or cluster, was then determined based on probability proportional to size of annual induced abortion and PAC caseload. In each facility, the enumerator used a systematic sampling technique to select and recruit every other eligible client in a one-month recruitment and interview period.

The sample size of clients was estimated using a single population proportion formula. The estimated number of women of reproductive age in the four regions at the time of data collection was 18,531,086 (32). Acknowledging that not everyone comes to public sector facilities when seeking abortion care, we purposely used the projected population size of reproductive-aged women in place of total women seeking facility-based services (i.e., public facility abortion caseload statistics) to increase the sample size, enhance the statistical power of our study, and ensure adequate representation of the target population. We calculated the sample size based on this projected population size and the assumption that 50% clients would report acceptable person-centered care with a precision that would produce a 95% confidence interval. We set a design effect of three as a multiplier to increase the sample size to account for the cluster effect of the study design and a 10% increase was included to account for non-response. The STATCALC function of Epi Info version 7 was used for this calculation, finding a target sample size of 1,152 CAC clients. During data collection, a one-month interview and recruitment period was set across all facilities to achieve the minimum sample size, rather than specific participant targets by site. This approach contributed to an unintentional protocol deviation caused by higher than predicted caseloads at each facility and led to interviewing 2,009 CAC clients, exceeding the target sample size.

### 2.3 Survey development

The client exit survey focused on the experience of CAC clients at the health facility before, during, and after their procedure. The survey covered CAC clients’ experience receiving timely care, having autonomy, with confidentiality, being treated respectfully, of discrimination or abuse, with the physical infrastructure of the health facility, and more. Questions included in the survey were adapted for CAC and to the Ethiopian context from a validated respectful maternity care questionnaire developed by Sheferaw et al. (33) and a health facility responsiveness questionnaire developed by the WHO (34). The original questionnaires were designed as scales to measure client experience of compassionate care and the responsiveness of health systems and facilities to patient needs. These evidence-based tools were used because the research was conducted before the development of a standardized and validated scale to measure the quality of abortion services. The data collection instrument for the client exit surveys was translated into the respective local languages of the study regions, including Amharic and Afan Oromo, and then back translated into English by independent translators. Local data collectors pre-tested the questionnaire, prior to data collection, through 20 pilot interviews at two hospitals and one health center. Based on the pilot study findings, the research team made appropriate amendments to the survey language and order of questions to improve flow and increase clarity.

### 2.4 Data collection and ethical considerations

Data collection procedures in this study were designed and conducted with attention to key ethical and quality considerations for participants, health facility staff, researchers, and all those involved in the data collection process. The data collectors consisted of health care workers outside of the sampled health facility who had at least a diploma in health sciences to ensure they had a base-level of knowledge regarding healthcare and working with patients and to increase participants willingness to respond honestly about their experience in the health facility. To establish high-quality and ethical data collection, there was a data collection orientation held in each study region. During this three-day orientation, all data collectors were trained on the research study, content in the questionnaire, navigating sensitive issues, informed consent, confidentiality, probing, in addition to other relevant study procedures and ethical considerations.

During data collection, supportive supervision was provided to data collectors to confirm accuracy and completeness of data. Data collectors followed all ethical guidelines including garnering written informed consent from participants, informing clients of the voluntary nature of the study, explaining benefits and risks of participation in the study and that participation in the study will not impact future health services. Considerations for participants safety and confidentiality, due to the sensitive nature of induced abortion and PAC, were incorporated including conducting interviews in a private setting inside the health facility and not collecting any identifiable information. Interviews were conducted in the language participants felt most comfortable with and were administered via a paper-based in-person survey. No remuneration was provided to participants following completion of the survey. Recruitment, consent, and interviews were all completed on the same day that participants received the abortion procedure, and all steps were conducted after the client received health services and before they left the facility.

### 2.5 Outcome development & data analysis

All survey data were entered into CSpro 7 and then exported to Stata version 14, where all data cleaning, exploration, and statistical analyses were conducted. We removed 132 participants with high levels of missing data, for a final sample of 1,870 study participants from 76 health facilities. Independent variables of interest included demographic characteristics (i.e., age, residence location, marital status, educational attainment), facility region (Oromia vs. Amhara vs. SNNP vs. Tigray), health facility type (health center vs. primary hospital vs. secondary hospital vs. tertiary hospital), diagnosis (induced abortion vs. PAC), and procedure type (MA vs. MVA).

Two scales adapted for this study setting and population were utilized in the questionnaire, therefore one of the initial steps in our data analysis process was conducting exploratory and confirmatory factor analysis (EFA and CFA) to test the structure of the respectful maternity care (33) and health facility responsiveness (34) scales for the Ethiopian context and abortion measurement. Factor analysis revealed poor scale validity and reliability of both for the Ethiopian abortion care context. Therefore, we decided to shift our focus to a secondary analysis of the collected data whereby we mapped individual items from the fielded questionnaire to outcome themes based on the domains of the Person-Centered Care Framework for Reproductive Health Equity (29), instead of using the originally-planned composite scale metrics.

First, outcomes collected in the questionnaire were thematically mapped to one of five person-centered care domains from the Person-Centered Care Framework for Reproductive Health Equity (29): dignity & respect; autonomy; communication & supportive care; trust, privacy, and confidentiality; and health facility environment (Table 1). Unfortunately, a sixth domain from the Person-Centered Care Framework for Reproductive Health Equity—that is, social support—was not included in the questionnaire and was therefore necessarily omitted from analysis. We discuss this limitation in the Discussion. At this stage, we decided to exclude other service quality outcomes collected that did not map to any of the five person-centered abortion care domains. In addition, outcomes with greater than 10% missing data were excluded from analysis. This led to the inclusion of 30 individual outcomes in our analysis. Table 1 presents the five person-centered care domains used in the analysis, as well as domain definitions and service outcomes for each domain to illustrate this mapping process.

**Table 1:** Person-Centered care framework for reproductive health equity domains, definitions, and corresponding outcomes.

Domain	Definition	Outcomes	Variable type
Autonomy	Autonomy refers to healthcare providers who respect women's views, support women to make educated decisions about their own care and obtain informed consent prior to procedures.	Were you given the opportunity to choose the type of abortion procedure that you received today?	Binary**
		How would you rate your experience of being involved in making decisions about your health care or treatment as much as you wanted?	Ordinal
		How would you rate your experience of being asked permission before performing any procedure or starting treatment?	Ordinal
Communication & supportive care	Communication & supportive care refer to healthcare providers providing timely and compassionate care through clear explanations of procedures, purpose of treatments, expected side effects, as well as integration of care that is responsive to patient needs. They confirm that women understand their explanations by using appropriate language for women to understand and ensuring patient care and safety.	Did you receive any pain medication before and after the procedure?	Binary**
		The health workers spoke to me in a language that I could understand.	Binary
		The health worker responded to my needs whether or not I asked.	Binary
		Received family planning counselling in addition to abortion procedure?	Binary**
		In your opinion, how do you describe the duration of your consultation with provider?	Binary*
		In your opinion how do you describe your wait time in the facility between the time you first arrived and the time you saw a provider?	Binary*
		How would you rate your experience of getting prompt attention at the health service?	Ordinal
		How would you rate your experience of getting enough time to ask questions about your health problem or treatment?	Ordinal
		How would you rate the experience of how clearly health care providers explained things to you?	Ordinal
		How would you rate your experience of getting information about other types of treatments or tests?	Ordinal
Trust, privacy, and confidentiality	Trust, privacy, and confidentiality refers to women's perceptions of competence in their healthcare providers and facility. Privacy refers to both the environment in which women's care is provided and during procedures/physical examinations and to ensuring medical records are kept confidential.	How would you rate the way your privacy was respected during physical examinations and treatments?	Ordinal
		How would you rate the way the health services ensured you could talk privately to health care providers?	Ordinal
		How would you rate the way your personal information was kept confidential?	Ordinal
Dignity & respect	Dignity & respect refer to the ability of women to receive care from their healthcare providers and other health facility staff in a respectful and caring setting. It captures typologies of physical and verbal abuse.	I felt that health workers cared for me with a kind approach.	Binary
		The health workers treated me in a friendly manner.	Binary
		All health workers treated me with respect as an individual.	Binary
		The health worker showed his/her concern and empathy.	Binary
		The provider called me by my name.	Binary
		The health provider scolded me during the procedure for different reason.	Binary
		The health workers shouted at me because I haven't done what I was told to do.	Binary
		Some of the health workers did not treat me well because of some personal attributes.	Binary
		Some health workers insulted me and my companions due to personal attributes.	Binary
		How would you rate your experience of being greeted and talked to respectfully?	Ordinal
		How would you rate your experience of being treated with respect and dignity?	Ordinal
Health facility environment	This captures the quality of the facility and providing a fully enabled environment, including the commodities and equipment, but also referral system, communication and transportation, maternal and neonatal health team that can cover the full continuum of care, environment where staff are respected, valued, and that is clean, and the extent to which a health facility offers a welcoming and pleasant environment. Examples include clean surroundings and enough space in waiting rooms and wards.	Did you pay any fee for the services you obtained in this facility?	Binary**
		How would you rate the cleanliness of the rooms inside the facility, including toilets?	Ordinal
		How would you rate the amount of space in the waiting and examination rooms?	Ordinal

Dignity & respect domain definition has been adapted to encompass care received from both providers and other facility staff. Communication & supportive care domain definitions has been adapted to include two relevant aspects: timeliness and integration of reproductive health services.

All ordinal variables were analyzed as three-level ratings with the answer categories good, moderate, and bad. All binary variables except those marked with * or ** were asked and analyzed as agree/disagree questions.

* Variables were originally asked in the survey as ordinal but were dichotomized with the answer categories satisfied/unsatisfied for analysis purposes.

** Variables were asked and analyzed with yes/no answer categories.

Second, we operationalized each of the 30 outcome definitions as either binary or ordinal outcomes to improve standardization and comparability across outcomes coming from different source scales. Specifically, questions adapted from the respectful maternity care questionnaire used a 5-point Likert scale with the following response categories: strongly agree, agree, don't know, disagree, strongly disagree. Due to the known limitations in interpreting “don't know” as the 3rd point of the Likert scale (35), we decided to exclude these responses (less than 7% of responses for all outcomes) from the analysis and collapse the remaining categories into binary variables: strongly agree and agree collapsed into one category and strongly disagree and disagree responses combined. Questions adapted from the health facility responsiveness questionnaire also used a 5-point Likert scale with very good, good, moderate, bad, very bad as the response options. We collapsed these outcomes into three-level ordinal variables with very good and good collapsing into a single category, moderate responses remaining in a moderate category, and combining very bad and bad into one category.

We calculated descriptive statistics for all independent variables and service quality outcomes. Bivariate analyses, including Pearson's chi square test and Kruskal Wallis tests, were conducted for all service quality outcomes by independent variables of interest (noted above) depending on how the outcome variable was operationalized (binary or ordinal). We conducted multivariable logistic regressions and ordered logistic regressions on each person-centered care outcome and the independent variables of health facility type, diagnosis, and procedure type. All multivariable regression models accounted for clustering by health facility and included the following independent variables: health facility type, age, marital status, educational attainment, diagnosis, and procedure type. We omitted the health facility region from the adjusted multivariable models because of limited variability. For example, facility region perfectly predicted success on a subset of outcomes, nullifying its utility as a control variable. For all levels of analysis, *p*-values less than 0.05 were considered statistically significant.

## 3 Results

### 3.1 Demographic and service characteristics of participants

**Figure 1:**
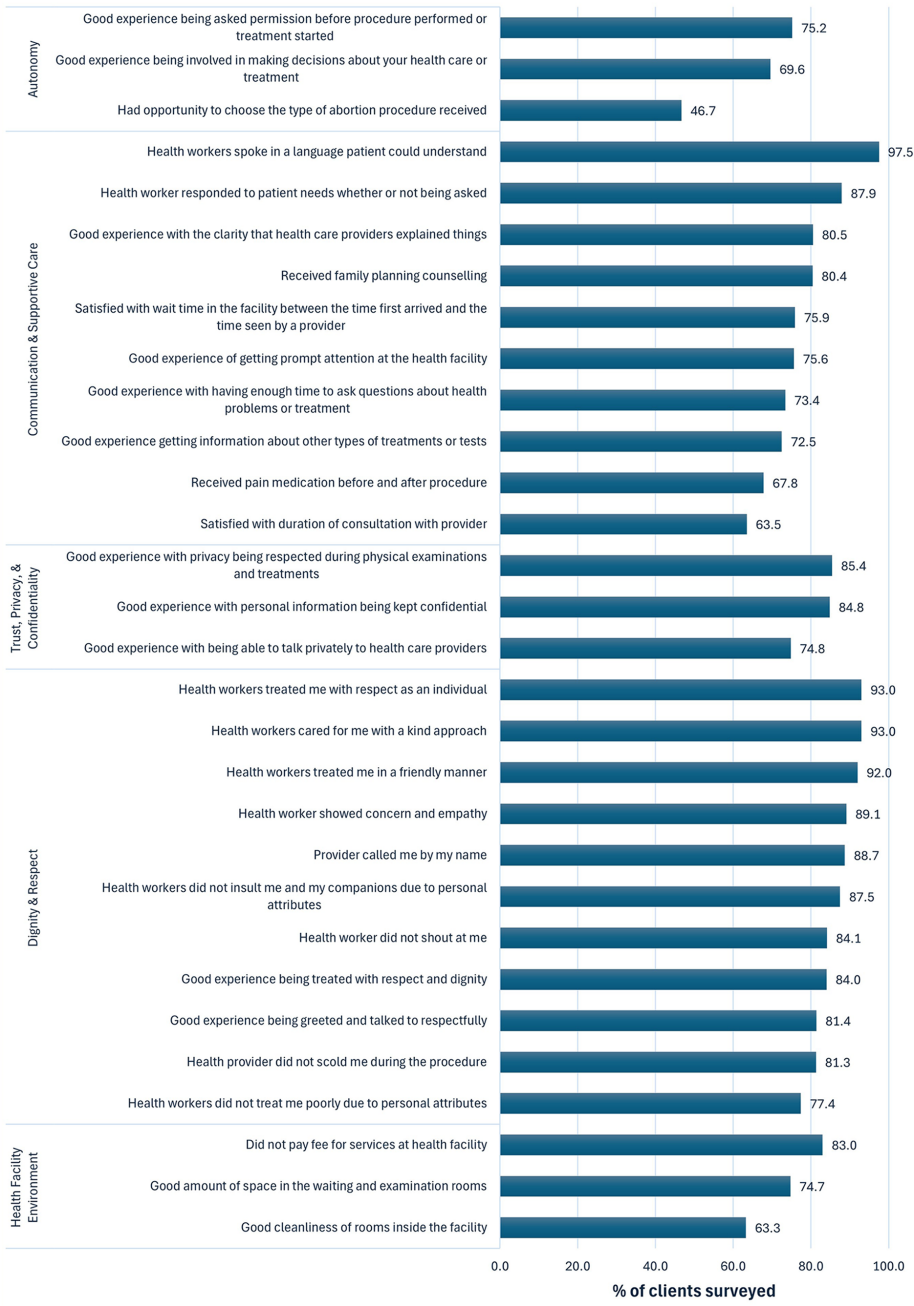
Outcomes by person-centered care domain (N=1,870).

Table 2 presents characteristics for the final sample of 1,870 CAC clients. Participants were aged 25.3 ± 6.2 years. Over one-third of respondents received care at secondary hospitals (35.9%), followed by tertiary hospitals (27.1%), primary hospitals (19.5%), and health centers (17.5%). Just over half of respondents (51.1%) were seeking induced abortion services. Just over half (51.7%) of participants received MA and 48.3% received MVA. Below, we present our findings by the five person-centered care domains (Figure 1), as well as disaggregated by the independent variables (Tables 3, 4).

**Table 2:** Socio-demographic and background characteristics of respondents (*n* = 1,870).

Background characteristics	n (%)^a^
Age (mean, median, sd)	(25.29, 24, 6.22)
18 and under	195 (10.5)
19–24	748 (40.3)
25 and over	913 (49.2)
Marital status	
Never married	729 (39.3)
Ever married	1,127 (60.7)
Educational level completed	
No formal education	810 (43.7)
Primary	547 (29.5)
Secondary or above	498 (26.8)
Residence	
Urban	1,301 (69.6)
Rural	569 (30.4)
Facility region	
Tigray	565 (30.2)
Amhara	451 (24.1)
Oromia	623 (33.3)
SNNPR	231 (12.4)
Health facility type	
Tertiary/comprehensive specialized hospital	507 (27.1)
Secondary/general hospital	671 (35.9)
Primary hospital	364 (19.5)
Health center	328 (17.5)
Reason for visiting facility	
Facility-based induced abortion care	941 (51.1)
For postabortion care	901 (48.9)
Type of procedure	
Evacuation using instrument (MVA)	870 (48.3)
Evacuation using tablet/pills (MA)	933 (51.7)

^a^ Percentages shown are among non-missing results; no variable had higher than 5% missing data.

**Table 3:** Bivariate analysis of person-centered care outcomes disaggregated by independent variables.

Person-centered care outcome		Health facility Type				Facility region				Diagnosis		Procedure type	
	All clients (*n* = 1,870)	Tertiary hospital (*n* = 211)	Secondary hospital (*n* = 944)	Primary hospital (*n* = 395)	Health center (*n* = 320)	Tigray (*n* = 565)	Amhara (*n* = 451)	Oromia (*n* = 623)	SNNPR (*n* = 231)	Safe induced abortion (*n* = 941)	Post-abortion care (*n* = 901)	Evacuation using instrument (*n* = 870)	Evacuation using tablet/pill (*n* = 933)
	n (%)	(%)	(%)	(%)	(%)	(%)	(%)	(%)	(%)	(%)	(%)	(%)	(%)
Autonomy													
Had opportunity to choose the type of abortion procedure received*^αβγΔ^*	853 (46.7)	30.8	47.0	45.1	72.9	47.9	26.9	62.0	40.5	62.7	29.9	33.3	59.2
†Good experience being involved in making decisions about your health care or treatment^αβγ*Δ*^	1,258 (69.6)	63.4	77.1	57.7	78.1	88.7	45.5	70.9	66.2	78.5	60.6	63.8	76.2
†Good experience being asked permission before procedure performed or treatment started^αβγ*Δ*^	1,359 (75.2)	69.2	81.8	66.1	81.8	91.3	55.9	74.0	76.1	83.8	66.4	70.6	79.4
Communication & supportive care													
Received pain medication before and after procedure^αβγ*Δ*^	1,237 (67.8)	50.6	74.0	76.7	72.5	68.1	61.8	68.9	75.4	62.1	74.0	76.9	59.7
Satisfied with duration of consultation with provider^αβ*Δ*^	1,149 (63.5)	70.3	64.2	55.4	60.3	65.7	62.1	56.0	80.5	64.8	61.7	61.3	65.8
Satisfied with wait time in the facility between the time first arrived and the time seen by a provider^αβγ*Δ*^	1,386 (75.9)	78.8	70.8	76.5	81.0	81.3	62.3	77.2	84.7	78.4	73.5	73.6	77.6
†Good experience of getting prompt attention at the health facility^αβγ^	1,398 (75.6)	73.3	77.7	70.1	81.2	89.5	58.6	71.2	86.5	80.1	71.1	74.2	76.5
†Good experience with having enough time to ask questions about health problems or treatment^αβγ*Δ*^	1,328 (73.4)	63.6	80.8	64.6	84.6	87.8	54.3	75.3	70.3	80.0	66.8	69.3	77.6
Health workers spoke in a language patient could understand^αβγ^	1,806 (97.5)	95.6	97.4	97.8	100.0	97.0	95.3	98.7	99.6	97.9	97.2	98.0	97.1
Health worker responded to patient needs whether or not being asked^αβγ*Δ*^	1,624 (87.9)	84.1	90.7	82.9	93.5	94.1	77.1	90.5	87.0	93.5	81.8	84.0	91.5
†Good experience with the clarity that health care providers explained things^αβγ*Δ*^	1,457 (80.5)	76.3	86.8	72.8	83.6	92.6	63.5	81.4	82.4	86.0	75.1	77.8	83.1
†Good experience getting information about other types of treatments or tests^αβγ*Δ*^	1,311 (72.5)	64.6	79.9	63.0	81.5	87.2	52.0	74.9	70.3	81.1	63.7	68.2	76.9
Received family planning counselling^αβγ^	1,456 (80.4)	69.7	84.1	78.0	91.9	81.0	66.5	89.7	80.4	82.8	78.1	79.5	81.3
Trust, privacy, & confidentiality													
†Good experience with privacy being respected during physical examinations and treatments^αβγ^	1,547 (85.4)	83.7	89.1	81.0	85.6	94.5	76.2	83.0	87.4	89.1	81.8	84.2	86.4
†Good experience with being able to talk privately to health care providers^αβγ*Δ*^	1,358 (74.8)	71.5	79.7	66.6	79.7	90.6	54.3	72.3	82.5	83.4	66.2	70.3	79.0
†Good experience with personal information being kept confidential^αβγ*Δ*^	1,540 (84.8)	80.9	87.6	79.9	91.2	94.7	75.6	80.3	90.0	90.2	79.1	82.1	87.4
Dignity & respect													
Health workers cared for me with a kind approach^β^	1,723 (93.0)	94.5	94.1	92.0	89.6	97.3	88.7	90.0	98.7	92.2	93.6	94.0	91.8
Health workers treated me in a friendly manner^β^	1,705 (92.0)	93.3	92.1	89.3	92.6	95.0	88.9	89.1	97.8	92.0	91.8	91.7	92.0
Health workers treated me with respect as an individual^αβγ^	1,725 (93.2)	93.1	93.8	90.9	94.8	94.5	88.4	94.6	95.7	94.1	92.1	92.8	93.6
Health worker showed concern and empathy^αβγ^	1,651 (89.1)	88.9	92.9	83.0	88.3	94.5	83.3	86.2	94.4	90.0	87.8	89.4	88.4
Provider called me by my name^αβγ*Δ*^	1,643 (88.7)	85.3	92.2	81.3	94.8	87.2	78.0	97.2	90.5	90.7	86.5	86.8	90.3
†Good experience being greeted and talked to respectfully^αβγ^	1,505 (81.4)	79.4	83.2	75.0	87.7	91.5	69.7	78.0	88.3	84.9	77.8	79.8	82.4
†Good experience being treated with respect and dignity^αβγ^	1,552 (84.0)	83.7	87.3	75.8	86.7	93.4	71.7	82.0	90.0	87.0	81.0	83.1	84.4
Health provider did not scold me during the procedure^α^	1,498 (81.3)	81.3	78.8	86.3	80.6	82.9	83.7	75.5	88.1	81.7	81.0	80.9	81.3
Health worker did not shout at me^αβ^	1,548 (84.1)	87.8	80.6	85.4	84.2	81.5	86.9	83.8	86.0	83.8	84.3	84.5	83.6
Health workers did not treat me poorly due to personal attributes^αβ^	1,418 (77.4)	83.9	76.3	78.7	68.0	81.3	71.5	73.1	90.4	77.7	76.6	77.8	76.7
Health workers did not insult me and my companions due to personal attributes^αβ^	1,619 (87.5)	90.5	85.6	87.0	87.4	84.2	88.6	87.2	94.4	88.4	86.7	87.3	86.9
Health facility environment													
†Good cleanliness of rooms inside the facility^αβγ*Δ*^	1,151 (63.3)	55.8	60.4	69.9	74.7	69.6	59.5	61.1	61.1	67.0	59.8	61.2	64.6
†Good amount of space in the waiting and examination rooms^β^	1,358 (74.7)	74.5	75.0	77.7	70.9	73.1	80.6	72.5	72.9	74.0	75.6	76.4	72.8
Did not pay fee for services at health facility^αβ^	1,478 (83.0)	81.7	79.4	82.0	93.1	81.7	86.1	91.8	54.8	81.5	84.8	84.4	81.7

All percentages shown are among non-missing data.

Outcomes noted with † are three-level ordinal variables with the categories good, moderate, and bad.

KEY: α by health facility type *p* < 0.05; β by region *p* < 0.05; γ by diagnosis *p* < 0.05; *Δ* by procedure type *p* < 0.05.

**Table 4:** Multivariable results of person-centered care outcomes.

Person-centered care domain	Person-centered care outcome	Regression model co-variates				
		Health facility type			Diagnosis	Procedure type
		Secondary hospital *AOR [95% CI]*	Primary hospital *AOR [95% CI]*	Health center *AOR [95% CI]*	Induced abortion care *AOR [95% CI]*	Evacuation using tablet/pills *AOR [95% CI]*
Autonomy	Opportunity to choose abortion procedure type	2.47 [1.06, 5.81]*	2.86 [1.31, 6.22]**	6.38 [2.29, 17.76]***	3.47 [2.30, 5.24]***	1.53 [1.06, 2.21]*
	^a^Experience being involved in making decisions about your health care	2.64 [1.10, 6.30]*	1.19 [0.44, 3.19]	2.12 [0.83, 5.44]	2.17 [1.39, 3.38]**	1.07 [0.70, 1.63]
	^a^Experience being asked permission prior to procedure or treatment	2.55 [1.09, 5.97]*	1.08 [0.41, 2.87]	1.68 [0.65, 4.35]	2.98 [1.89, 4.71]***	0.86 [0.59, 1.25]
Communication & supportive care	Received pain medication	2.74 [1.01, 7.43]*	3.26 [1.35, 7.87]**	3.00 [1.09, 8.22]*	0.80 [0.45, 1.41]	0.49 [0.32, 0.77]**
	Duration of consultation	0.81 [0.34, 1.91]	0.62 [0.28, 1.34]	0.70 [0.32, 1.58]	1.04 [0.65, 1.69]	1.16 [0.76, 1.78]
	Wait time between arriving and being seen by provider	0.66 [0.27, 1.61]	1.02 [0.49, 2.11]	1.17 [0.47, 2.92]	1.44 [0.96, 2.16]	1.03 [0.72, 1.48]
	^a^Experience receiving prompt attention	1.42 [0.57, 3.54]	1.06 [0.38, 2.94]	1.74 [0.71, 4.24]	2.24 [1.33, 3.76]**	0.76 [0.51, 1.15]
	^a^Experience of having time to ask questions	2.93 [1.27, 6.78]*	1.38 [0.59, 3.22]	3.00 [1.12, 8.03]*	1.92[1.20, 3.10]**	1.06 [0.70, 1.62]
	Understood language used by health workers	3.37 [1.23, 9.22]*	3.27 [1.29, 8.26]*	n/a	3.49 [1.59, 7.68]**	0.29 [0.11, 0.77]*
	Health worker responsive to patient needs	2.51 [0.79, 7.95]	1.65 [0.59, 4.62]	3.04 [0.90, 10.21]	2.29 [1.23, 4.27]**	1.17 [0.59, 2.31]
	^a^Experience of clear communication from provider	2.23 [0.94, 5.52]	1.00 [0.38, 2.64]	1.46 [0.53, 3.94]	2.29 [1.35, 3.87]**	1.00 [0.63, 1.60]
	^a^Experience receiving information about other treatments/tests	2.88 [1.19, 6.96]*	1.26 [0.47, 3.39]	2.24 [0.87, 5.75]	2.91 [1.66, 5.09]***	0.84 [0.57, 1.23]
	Received family planning counselling	2.44 [0.92, 6.46]	1.87 [0.60, 5.83]	4.86 [1.36, 17.35]*	1.35 [0.74, 2.48]	0.84 [0.58, 1.23]
Trust, privacy & confidentiality	^a^Experience of having physical privacy respected	1.73 [0.65, 4.62]	0.92 [0.31, 2.77]	1.09 [0.38, 3.12]	2.46 [1.15, 5.25]*	0.85 [0.50, 1.44]
	^a^Experience of talking privately to health care providers	1.90 [0.76, 4.75]	1.03 [0.38, 2.79]	1.38 [0.47, 4.06]	2.99 [1.78, 5.03]**	0.82 [0.55, 1.24]
	^a^Experience of having personal information kept confidential	1.96 [0.68, 5.61]	1.12 [0.36, 3.51]	2.07 [0.52, 8.19]	3.31 [1.68, 6.53]**	0.88 [0.51, 1.50]
Dignity & respect	Treated with kind approach	1.87 [0.61, 5.74]	0.86 [0.17, 4.32]	1.00 [0.21, 4.89]	1.65 [0.70, 3.92]	0.89 [0.54, 1.48]
	Treated in a friendly manner	1.83 [0.67, 4.99]	0.95 [0.30, 3.01]	1.23 [0.33, 4.63]	1.41 [0.67, 2.96]	0.92 [0.51, 1.66]
	Treated with respect	2.51 [1.01, 6.28]*	1.02 [0.28, 3.69]	1.70 [0.37, 7.73]	2.06 [0.89, 4.77]	0.83 [0.54, 1.27]
	Shown concern and empathy	2.51 [1.03, 6.12]*	0.90 [0.27, 2.99]	1.76 [0.40, 7.84]	1.96 [0.97, 3.95]	0.86 [0.49, 1.51]
	Provider called me by my name	2.80 [0.92, 8.58]	0.64 [0.14, 2.91]	2.67 [0.57, 12.60]	1.62 [0.87, 3.01]	1.10 [0.69, 1.77]
	^a^Experience being greeted and talked to respectfully	1.38 [0.62, 3.11]	0.87 [0.36, 2.12]	1.81 [0.72, 4.55]	1.90 [1.20, 3.03]**	0.91 [0.61, 1.35]
	^a^Experience being treated with dignity and respect	1.45 [0.61, 3.44]	0.67 [0.26, 1.71]	1.23 [0.46, 3.31]	1.95 [1.18, 3.24]*	0.79 [0.54, 1.15]
	Health provider scolded me	1.02 [0.31, 3.31]	0.45 [0.17, 1.18]	0.83 [0.33, 2.10]	0.78 [0.50, 1.23]	1.06 [0.66, 1.69]
	Health worker shouted at me	1.50 [0.46, 4.92]	0.68 [0.32, 1.47]	1.10 [0.49, 2.48]	0.89 [0.54, 1.47]	1.26 [0.80, 1.99]
	Not treated well because of personal attribute	1.78 [0.51, 6.20]	1.10 [0.35, 3.44]	2.04 [0.78, 5.36]	0.81 [0.42, 1.54]	1.08 [0.65, 1.81]
	Insulted me and my companions because of personal attributes	1.72 [0.37, 7.95]	1.12 [0.26, 4.90]	1.44 [0.53, 3.93]	0.62 [0.29, 1.33]	1.37 [0.88, 2.14]
Health facility environment	^a^Health facility cleanliness	1.13 [0.41, 3.09]	1.72 [0.52, 5.67]	2.14 [0.67, 6.80]	1.37 [0.82, 2.31]	0.98 [0.66, 1.44]
	^a^Health facility space	0.99 [0.33, 3.03]	1.07 [0.33, 3.46]	0.83 [0.21, 3.26]	1.18 [0.69, 2.00]	0.88 [0.62, 1.27]
	Paid fee for services	1.24 [0.35, 4.37]	0.97 [0.29, 3.22]	0.33 [0.08, 1.32]	1.44 [0.78, 2.67]	1.16 [0.68, 2.00]

Controlled for age, marital status, and education level.

Reference categories: tertiary hospitals, post-abortion care, and evacuation with instrument.

^a^ three-level ordinal variable with good, moderate, bad categories.

*p < 0.05.

**p < 0.01.

***p < 0.001.

### 3.2 Autonomy

Participants indicated low levels of autonomy, with over half (53.3%) reporting they were unable to choose their procedure type and nearly one-third (30.4%) rating their involvement in making decisions about their own health care as bad or moderate. However, three-quarters (75.2%) of CAC clients reported that they had a good experience with being asked permission before any procedure was started (Figure 1).

Only 30.8% of CAC clients at tertiary hospitals were able to choose their procedure method compared to 72.9% of those at health centers (Table 3). CAC clients at health centers (AOR = 6.38, *p* < 0.001), primary hospitals (AOR = 2.86, *p* < 0.001), and secondary hospitals (AOR = 2.47, *p* < 0.05) all had higher odds of having the chance to choose their procedure type compared to individuals who received abortion services in tertiary facilities (Table 4). CAC clients who received services at secondary hospitals had higher odds of reporting a good experience with health care decision making (AOR = 2.6, *p* < 0.05) and being asked for permission prior to procedure (AOR = 2.6, *p* < 0.05) when compared to tertiary facilities.

Induced abortion clients had increased odds (AOR = 3.5, *p* < 0.001) of being able to choose their procedure type compared to PAC clients. Specifically, only 29.9% of PAC clients were able to choose their procedure type compared to 62.7% of safe induced abortion clients (Table 3). Induced abortion clients also had higher odds of being involved in personal health care decisions (AOR = 2.2, *p* < 0.01) and being asked permission prior to procedure (AOR = 2.98, *p* < 0.001) than PAC clients. Lastly, respondents who received MA had increased odds of being able to choose their procedure type compared to MVA clients (AOR = 1.5, *p* < 0.05).

### 3.3 Communication & supportive care

We found high levels of clear communication and supportive care, with 97.5% of respondents agreeing that their health provider spoke in an understandable language and 87.9% indicating that their provider responded to their needs. In contrast, approximately one-quarter of respondents indicated dissatisfaction with their wait time (24.1%) and a moderate or bad experience receiving prompt attention at the facility (24.4%). Over one-third (36.5%) of CAC clients were unsatisfied with the duration of their consultation time, and over one-quarter (26.6%) rated their amount of time to ask their provider questions as bad or moderate. Over two-thirds (67.8%) of CAC clients received pain medication, 72.5% rated their experience getting information about other services as good, and 80.4% received family planning (FP) counselling.

Clients at health centers (AOR = 3.0, *p* < 0.05) and secondary facilities (AOR = 2.9, *p* < 0.05) were three times more likely than those at tertiary facilities to have a positive experience with enough time to ask their provider questions. Health center clients also had 4.9 higher odds of receiving FP counselling (*p* < 0.05) compared to those at tertiary facilities, with 91.9% receiving FP counselling compared to just 69.7%, respectively. Compared to respondents at tertiary hospitals, those at a secondary hospital were more likely to report a positive experience receiving information about other tests and treatments (AOR = 2.9, *p* < 0.05). The adjusted model also elucidated higher odds of receiving pain medication at health centers (AOR = 3.0, *p* < 0.05), primary hospitals (AOR = 3.2, *p* < 0.01) and secondary hospitals (AOR = 2.74, *p* < 0.05). In fact, only 50.6% of respondents who received care at a tertiary hospital reported receiving pain medication (Table 3).

Induced abortion clients reported better communication and supportive care compared to PAC clients across all significant outcomes in the multivariable analysis. Induced abortion clients had 2.3 times higher odds of reporting that the provider responded to their needs (*p* < 0.01), 2.2 times higher odds of being more likely to receive prompt attention (*p* < 0.01), and 2.3 times higher odds of being more likely to receive clear explanation of the treatment or procedure from their health care provider (*p* < 0.01). Additionally, they were more likely to indicate a good experience with having enough time to ask questions about health problems (AOR = 1.9, *p* < 0.01) and getting information about other services (AOR = 2.9, *p* < 0.001). With a couple notable exceptions, we found no significant variation in communication and supportive care by procedure type. At all facility types, MA clients had lower odds of receiving pain medication compared to MVA clients (AOR = 0.49, *p* < 0.01).

### 3.4 Trust, privacy, & confidentiality

Most CAC clients reported positive experiences with confidentiality. Over 4 in 5 (84.8%) respondents rated a good experience with their personal information being kept confidential. Only 14.6% of clients reported a bad experience with their privacy being respected during physical examinations and treatments, while 85.4% responded good for this outcome.

There were no significant differences in the multivariable analysis seen for the three outcomes in this domain by health facility type or procedure type. However, induced abortion clients reported better privacy and confidentiality compared to PAC clients, including physical privacy during procedure (AOR = 2.5, *p* < 0.05), talking privately with their provider (AOR = 2.99, *p* < 0.01), and confidentiality (AOR = 3.3, *p* < 0.001). In fact, 83.4% of induced clients had good experiences with their time speaking privately with a provider, while less than two-thirds (66.2%) of PAC clients reported the same (Table 3).

### 3.5 Dignity & respect

Nearly all CAC clients reported that the health provider used a kind approach (93.0%) and treated them in a friendly manner (92.0%) with respect (93.2%). Similarly, 89.1% of respondents reported that they were shown concern and empathy. However, a low but notable percent of CAC clients, 18.6% and 16.0% respectively, reported a moderate or bad experience being talked to respectfully and being treated with dignity. While a considerable majority of CAC clients did not experience instances of discrimination or abuse, 15.5% reported being scolded by a provider and 15.9% stated that they were treated poorly due to personal attributes. Slightly less indicated that they were shouted at by a provider (12.3%) or that their provider insulted them based on personal characteristics (10.6%).

CAC clients at secondary hospitals had 2.51 higher odds of being treated with respect (*p* < 0.05) and shown empathy (*p* < 0.05), compared to those at tertiary facilities. Induced abortion clients were more likely to report a good or moderate experience being talked to respectfully (AOR =  1.9, *p* < 0.01) and treated with respect and dignity (AOR = 1.95, *p* < 0.05). There were no significant associations identified in the multivariable analysis by procedure type for the outcomes related to dignity and respect.

### 3.6 Health facility environment

Overall, nearly three quarters (74.7%) of CAC clients in the study rated the spaces in the waiting room and examination rooms as good. Over one third (36.7%) of participants reported bad or moderate cleanliness of the procedure room. Although abortion in the public sector is free in Ethiopia, 17% of respondents paid for services received at the health facility. There were no significant associations identified between health facility environment outcomes and facility type, diagnosis, nor procedure type, in the multivariable analysis. Despite no differences identified across sub-groups, overall performance on health facility environment outcomes was consistently lowest among the person-centered care domains.

### 3.7 Regional variations in person-centered care

Twenty-nine of the 30 person-centered care outcomes were found to be significantly different by facility region in the bivariate analysis (Table 3). For 24 of the 29 outcomes (82.7%) where region was significant, those who received care in Amhara reported the poorest experience among the four regions. In fact, this trend was seen for all outcomes in the autonomy domain and the trust, privacy, and confidentiality domain. Amhara also had the lowest levels for nine of the ten communication and supportive care outcomes and eight of the eleven outcomes in the dignity and respect domain.

## 4 Discussion

### 4.1 Key findings

Overall, high levels of person-centered care were reported among all surveyed clients. Applying threshold guidance from indicators included in the Abortion Care Quality (ACQ) Tool (36), for the majority of outcomes (17 out of 30 outcomes), over 80% of the sample reported a positive experience. This is consistent with research from Addis Ababa, which found that people who received CAC in public facilities experienced high levels of satisfaction on person-centered care indicators similar to those in this study (28). The overall high quality of person-centered abortion care in Ethiopia is consistent with health experts’ consensus that liberal abortion policies and reduced institutional restrictions lead to improved CAC quality (12, 20).

The results also point to areas of person-centered abortion care in Ethiopian public health facilities that need improvement: autonomy, communication and supportive care, and health facility environment. Prior research supports focusing attention and resources to these components of CAC in Ethiopia and other countries. Specifically, induced abortion clients from Kenya and India emphasized interpersonal interactions with providers and health facility personnel as one of the most critical components of good quality abortion services—aligning well with the outcomes included in both the communication and supportive care and autonomy domains (37). Mossie Chekol et al. (28) identified interpersonal communication, receiving information related to the procedure, and the physical environment as three focus areas to improve CAC client satisfaction in Addis Ababa, corroborating our findings. Our results build upon these prior findings by expanding the analysis to other regions of Ethiopia.

The Abortion Care Guideline from WHO indicates that regardless of whether a client receives PAC or induced abortion services, all abortion clients deserve the same high quality of person-centered care (12). Consistent with previous studies (4), we found a high rate, nearly half, of clients seeking PAC services, despite induced abortion being available and accessible in the public sector (3, 5, 7). While prior research in Ethiopia has not found differences in the quality of client experiences between PAC and induced abortion services (28), our findings illuminate disparities between diagnosis categories, with induced abortion clients reporting higher levels of autonomy, communication and supportive care, as well as privacy and confidentiality than PAC clients. We hypothesize this may be indicative of the more serious and sometimes urgent nature of PAC services compared to induced care, but these differences warrant further investigation.

Consistently, our regional analysis indicated that CAC services in the Amhara region had the lowest levels of person-centered care across all domains. These results are consistent with a study which found that Amhara had the lowest family planning quality score and that there were only slight differences in family planning quality scores observed between the other regions studied (38). The identified regional disparities highlight the importance of evaluating person-centered care across multiple geographies to uncover potential disparities in quality and foster cross-regional learning.

CAC clients had higher levels of autonomy, communication, and supportive care at health centers and secondary facilities, than at tertiary hospitals. Baynes et al. (29) similarly concluded that the strongest predictor of high client satisfaction in Tanzania's public sector was related to facility type, with PAC clients more satisfied with services at lower-level facilities like health centers, than tertiary facilities. Lower-level facilities are often assumed to be understaffed and under resourced leading to the conclusion that they are unable to provide high-quality care (25, 39); our findings challenge this assumption and are consistent with primary care facilities in lower- and middle- income countries being effectively leveraged to provide high-quality HIV care and treatment (25). Similarly, the lowest rates of family planning counselling and having a good experience getting information about other health services were observed at primary and tertiary hospitals, with the highest rates seen at health centers. Wake et al. (40) demonstrated the importance of focusing on the integration of reproductive health services through analysis showing that postabortion contraception acceptance in Ethiopia is directly associated with increased family planning counselling.

Across all domains, very few disparities in person-centered care between CAC clients who received MVA or MA were identified. This conflicts with prior studies in Addis Ababa and neighboring Kenya, all which found significantly different levels of satisfaction and person-centered care by abortion procedure type. In these studies, MVA clients received better person-centered abortion care than MA clients (28, 41). One outcome, however, was consistent with these prior findings; MA clients were significantly less likely to receive pain medication than MVA clients. Pain is important to consider for MA as it is commonly noted as a reason for dissatisfaction among abortion clients (42). Less pain management among MA clients also conflicts with WHO guidelines which explicitly recommend that MA clients at any gestational age are offered pain management (12). There may be misconceptions among abortion clients related to pain and side effects of MA, potentially indicating a gap in pre-procedure counselling. In fact, a study in Northwest Ethiopia found that half of women selected MA over MVA as a way to avoid pain and therefore called for improved counselling on side effects and pain management (43). Kapp and colleagues also found that over one-third of women (37.4%) who received MA after 13 weeks gestation at an Addis Ababa hospital experienced more pain than they expected (44).

### 4.2 Strengths and limitations

This study had limitations that are important to note. First, the adapted scales used in the survey failed to pass confirmatory factor analysis and validity testing for the Ethiopian context. We addressed this limitation by analyzing each outcome individually rather than using a composite measure. An additional limitation was the omission of outcomes related to the sixth domain of person-centered care, social support, due to gaps in the questionnaire used and a lack of validated tools at the time of data collection. Furthermore, the context in Northern Ethiopia has changed drastically since data collection for this study due to the COVID-19 pandemic (45) and the conflict in Tigray. Health facilities and services across Northern Ethiopia have been devastated (46, 47). In fact, as of June 2021 reports indicate that only 13.5% of all health centers and hospitals were operating in the Tigray region, of course having a distressing impact on access and availability of SRH services, including induced abortion services and PAC (32, 48). This change in context has likely impacted the accuracy of our findings compared to the current state of abortion services in the four study regions of Ethiopia. Yet, the unique timing of this research provides a snapshot of the quality of CAC services in Tigray and the surrounding regions that can be used to benchmark future research and service quality monitoring as the region recovers from the humanitarian crisis and works to reestablish high quality CAC services in the local health system. Lastly, known limitations of client exit surveys for those seeking CAC include social desirability bias, low expectations of quality, and universally high satisfaction rates must be considered in interpretation of findings.

This study also had a variety of strengths. First, this research fills a recognized gap in the literature by focusing on person-centered care in public health facilities using client exit surveys. Second, this study explores person-centered abortion care using independent variables that few studies in Ethiopia or East Africa have used in the past, including by region and level of public health facility. Even studies which have obtained data from multiple regions in the country or multiple facility levels have not conducted analysis or disaggregation of data by these categories (23, 49, 50). Regional and facility considerations are important for localizing CAC quality improvement priorities, policies, and programs (23, 38).

### 4.3 Program and research implications

Our analysis highlights the need for concentrating quality improvement efforts on specific domains of person-centered abortion care, populations, and settings to target areas where there is the most opportunity for impact. It is critical for programs aiming to improve CAC client experiences to have components dedicated to increasing the autonomy of people seeking induced abortion or PAC services, improving the level of communication and supportive care from health care providers, and for addressing and preventing instances of abuse and discrimination experienced by CAC clients. Although CAC clients in our study reported nearly universally good experiences of dignity and respect, any instance of abuse or discrimination should not be tolerated as it constitutes a human rights violation (12). Therefore, although less than one in six CAC clients experienced being scolded, shouted at, discriminated, or insulted due to personal attributes, critical attention must be given to address this issue. Further, the low satisfaction reported among CAC clients related to the health facility environment pinpoints an additional opportunity for intervention and resource dedication at the policy level.

Specific program implications are clear from this study's key findings at facility and regional levels. Due to the high rates of PAC in our sample, programmatic efforts to reduce disparities between induced abortion care and PAC service quality is critical. Our results also may indicate the need for the development of guidelines and refresher trainings for providers on appropriate pain management and counseling for all types of abortion procedures, particularly MA. Additionally, concentrated initiatives are needed to improve CAC service quality at primary and tertiary hospitals with a specific focus on better integration of reproductive health services including family planning counselling. Based on our findings, contextual knowledge, and analysis of prior research, continuing to invest in task-sharing initiatives, within higher-level facilities, may be an effective intervention for regional and national health officials to consider, as an approach for both expanding access to CAC and improving client experiences (5, 51, 52). Importantly, due to the quantitative nature of this study, qualitative inquiry and direct observation research that integrates the perspectives of both abortion clients and providers would provide useful insight into the disparities in person-centered care we identified by diagnosis, facility level, and procedure type.

National-level actors can utilize the key findings from our analysis as a basis for improving monitoring and evaluation of CAC service quality. The results highlighted important person-centered care disparities in Amhara, in primary and tertiary hospitals, and among PAC clients, providing justification for future quality improvement efforts to include analysis of person-centered abortion care in Ethiopia and surrounding areas by health facility level, region, and diagnosis. Specifically, we recommend national health management information system (HMIS) integration and stakeholder adoption of indicators from the new ACQ Tool, released in 2022 (36, 53). A key strength of this tool is the intentional development of indicators that are client-centered, simple, effective, and tested in the Ethiopian context (53). Additionally, future person-centered care research should include measures of social support received by abortion clients using validated tools such as the Received Social Support Scale (54). We therefore recommend application of these tools for future investigations of person-centered abortion care in Ethiopia and beyond.

In this study, we aimed to evaluate the extent of person-centeredness experienced by CAC clients when seeking care at a public health facility in four regions of Ethiopia. In doing so, we build upon the existing person-centered abortion care literature in East Africa and identify key focus areas for future research efforts as well as, facility- and regional-level programs to improve the quality of CAC services in this context. Quality improvement efforts should concentrate on improving CAC clients’ autonomy, communication and supportive care, and the health facility environment. Relevant actors must dedicate resources to improve PAC quality, integration of reproductive health services with CAC, and pain management for MA clients as vital interventions for improving person-centered abortion care in public health facilities across Ethiopia.

## Data Availability

The raw data supporting the conclusions of this article will be made available by the authors, without undue reservation.

## Footnotes

^1^Postabortion care (PAC) includes the treatment of incomplete or unsafe abortions and any related complications.

^2^CAC is defined by the World Health Organization as the provision of information, abortion management (including induced abortion and care related to pregnancy loss), and PAC. We use the term CAC to describe a combined category of both facility-based induced abortion and PAC services/clients throughout this paper.

^3^“Women-centered abortion care” is defined as a “comprehensive approach to providing abortion services that takes into account the various factors that influence a woman's individual mental and physical health needs as well as her ability to access services and her personal circumstances and her ability to access services” (3). “Women” is the term used in the Ethiopian guidelines and study protocol. We acknowledge that women are not the only population who need and have the right to comprehensive abortion care; therefore, the term person will be used throughout this manuscript when possible. However, the term women will be used at times due to the cultural context, terminology utilized in the existing literature, and to accurately represent those included in the study population.
